# The Fever at San Antonio

**Published:** 1903-12

**Authors:** J. V. Spring


### The Fever at San Antonio.

BY DR. J. V. SPRING.

*Editor Texas Medical Journal:*

The purpose of this paper is to clear up the misunderstanding which has unfortunately occupied the public mind in regard to yellow fever in San Antonio; to give a statement of conditions just as they have been from the report of the first "suspicious case" to the present time, which can be supported by affidavits, if necessary.

A man should fully realize the responsibility resting upon him when he accepts a public office. Especially is this true when a physician assumes the relation of advisor, and so far as professional subjects are concerned, the guide, to those who legislate or execute the laws connected with public health. He may expect to be held responsible for the position which he takes, and advice that he gives, in regard to all public health matters. He should be thoroughly familiar with the conclusions of modern science with regard to any subject he may be called to pass upon; and when infectious and contagious diseases are to be considered, he should be familiar with the etiology, clinical history, pathology, diagnosis and treatment, and especially should he be well informed as to all points of differential diagnosis.

There is no question that at *this* time, in the minds of many intelligent physicians in San Antonio, there is a doubt as to the possibility of there having been yellow fever in our city at all. This doubt has been created by the history and management of reported cases, and confirmed by the failure of the infection to spread under the most favorable atmospheric conditions, and lack of necessary precautions being adopted.

When the first cases were reported, many were disposed to admit the possibility of one or more cases having been imported here from our Mexican border; and were unwilling to reject the opinions of eminent experts, who had declared certain cases to be genuine yellow fever. But when we consider the fact, that there has never been a single case arising from contact with, or mosquitoes from any other case of the twenty-two reported by the State Health Officer and local board of health, while we had twenty-two points of infection reported (each case declared to be a "typical case of yellow fever"), widely separated, in thickly populated districts, yet no new cases have been found in any of these houses, or neighborhoods, every new case reported in a new section, far removed from any other typical (?) case, and no evidence can be adduced that they had ever been to any who were sick (or reported sick) with yellow fever; and affidavits can be had to prove that many of those cases were not managed according to the rules laid down by the United States Marine Hospital Service, such as screens, mosquito bars, etc., or other precautions enforced to prevent the spread of this infection by mosquitoes or fomites, there is no great wonder that there should exist an honest doubt as to the character of the fever which has been found all over Southwest and Southeast Texas this fall.

The mosquito theory of transmission is of very recent origin, and as yet has not been accepted as the only means of infection or transmission from man to man, by many able and experienced physicians. Those who do not accept the mosquito theory, and who have lived in malarial districts where both malaria and yellow fever have prevailed in epidemic form, who have had personal experience in the diagnosis and management of both forms, are fully persuaded that the large majority of the cases in our city diagnosed yellow fever, have been cases of malarial fever described by authors as "asthenic," adynamic and "pernicious types of malarial fever."

Those who claim these cases to have been typical cases of yellow fever, lay great stress upon certain points of diagnosis, (1) the lack of correlation of pulse and temperature, (2) albumin in the urine, (3) jaundice, (4) irritable stomach and pain upon pressure in epigastric region, (5) hemorrhagic diathesis (nose bleed, bleeding gums, hæmaturia, etc.)

Let us see what some of the best authors say about these symptoms and conditions:

Prof. S. M. Bemis of New Orleans, La., says: "The slow pulse which coexists with elevated temperature is a point of diagnostic value, *but it must* be remembered that this symptom *is not* peculiar to yellow fever. I have noticed this lack of correlation of pulse and temperature in cases of dengue, and it is frequently found in ordinary cases of jaundice."

Dr. William Osler of Johns Hopkins' Hospital, says: "The presence of albumen in the urine upon which some writers lay such stress as a distinguishing feature in yellow fever, is far too common a symptom in all forms of malaria to be worth much as a guide." Again he says: "Yellow fever simulates closely, and may be mistaken for ordinary malarial fever of remittent type, and vice versa."

Dr. Adolf Strumpell, director of medical clinic at Erlanger, says: "In mild cases the symptoms *are not* distinctive, and the diagnosis not likely to be reached except by an experienced observer, and, even by him, more or less conjecturally. The only disease which can well give rise to confusion is remittent with jaundice." He also states that, "fear, anxiety, worry, or anything which tends to depress the nervous system, increases the danger." I would like to ask, what could create more fear, anxiety, worry, or nervous depression that to have a troop of experts (?) visit a sick room and have them discuss the symptoms, and declare the patient sick with such a dread scourge as yellow fever? I think that one or more of the fatal cases in our recent epidemic (?) was probably due to this cause.

In his description of remittent fever, Dr. Stumpell says: "The symptoms are those of a severe constitutional infection; gastro-intestinal symptoms may predominate, there may be jaundice, hæmaturia, and even general hæmorrhagic diathesis; sometimes the urine has considerable albumen. Genuine nephritis is met with in graver varieties of the disease."

Dr. Austin Flint, who had large experience with both yellow and malarial fevers, says, on diagnosis: "The access of the disease and symptoms *during the febrile stage* present nothing distinctive; all observers agree that it is often difficult to arrive at a positive diagnosis." In his clinical history of remittent fever he says: "Early in the fever nausea and vomiting *generally occur,* and are frequently prominent symptoms; pain and uneasiness is usually referred to the region of the stomach, and there is tenderness over the epigastrium,—the urine is scanty, often albuminous, and jaundice appears in a certain proportion of cases. *This disease differs much in severity at different times and places, and in different cases at the same time and place.*"

Let me say, in conclusion, that these twenty-two cases of alleged yellow fever covered a period of twenty-eight days in a city of 60,000 people, who were advised that "There is no danger; let the fair go on; keep your schools, theaters and churches open; do not worry." The only thing done to prevent the spread of this infection, outside of cleaning up an already clean city, was *State quarantine.* I would like to ask, can *any* city in our State show a cleaner bill of health during the time of our infection (?) than San Antonio?

